# Gene Expression Profiling of U12-Type Spliceosome Mutant *Drosophila* Reveals Widespread Changes in Metabolic Pathways

**DOI:** 10.1371/journal.pone.0013215

**Published:** 2010-10-11

**Authors:** Heli K. J. Pessa, Dario Greco, Jouni Kvist, Gudrun Wahlström, Tapio I. Heino, Petri Auvinen, Mikko J. Frilander

**Affiliations:** 1 Institute of Biotechnology, University of Helsinki, Helsinki, Finland; 2 Department of Biosciences, University of Helsinki, Helsinki, Finland; Centre de Regulació Genòmica, Spain

## Abstract

**Background:**

The U12-type spliceosome is responsible for the removal of a subset of introns from eukaryotic mRNAs. U12-type introns are spliced less efficiently than normal U2-type introns, which suggests a rate-limiting role in gene expression. The *Drosophila* genome contains about 20 U12-type introns, many of them in essential genes, and the U12-type spliceosome has previously been shown to be essential in the fly.

**Methodology/Principal Findings:**

We have used a *Drosophila* line with a P-element insertion in U6atac snRNA, an essential component of the U12-type spliceosome, to investigate the impact of U12-type introns on gene expression at the organismal level during fly development. This line exhibits progressive accumulation of unspliced U12-type introns during larval development and the death of larvae at the third instar stage. Surprisingly, microarray and RT-PCR analyses revealed that most genes containing U12-type introns showed only mild perturbations in the splicing of U12-type introns. In contrast, we detected widespread downstream effects on genes that do not contain U12-type introns, with genes related to various metabolic pathways constituting the largest group.

**Conclusions/Significance:**

U12-type intron-containing genes exhibited variable gene-specific responses to the splicing defect, with some genes showing up- or downregulation, while most did not change significantly. The observed residual U12-type splicing activity could be explained with the mutant U6atac allele having a low level of catalytic activity. Detailed analysis of all genes suggested that a defect in the splicing of the U12-type intron of the mitochondrial *prohibitin* gene may be the primary cause of the various downstream effects detected in the microarray analysis.

## Introduction

The removal of noncoding introns by the spliceosome is an essential step in the posttranscriptional processing of mRNAs in eukaryotic organisms. Most multicellular organisms, including plants, vertebrates and insects, and some unicellular eukaryotes have two distinct spliceosomes that remove divergent intron types. In addition to the ubiquitous U2-type “major” spliceosome that removes the majority of all introns, the U12-type “minor” spliceosome excises a subset of introns containing highly conserved 5′ splice site (5′ss) and branch point sequences (BPS) [1], [2], [3], [4].

Both spliceosomes use a similar mechanism for intron removal and consist of five small nuclear RNAs (snRNA) assembled into ribonucleoprotein (snRNP) particles containing more than 150 different protein species (for reviews, see [5], [6]). The snRNA composition differs between the two systems. The specific snRNAs of the U12-type spliceosome are U11, U12, U4atac and U6atac, and the functional analogs in the major spliceosome are U1, U2, U4 and U6, respectively [7], [8], [9]. U5 snRNA and most protein components are shared between the two spliceosomes [8], [10], but at least the mammalian system utilizes seven protein species specific to the U12-dependent spliceosome [11].

Splicing of U12-type introns begins with a cooperative recognition of both the 5′ss and the BPS by the U11/U12 di-snRNP [7], [8], [12], [13], followed by the entry of the U4atac/U6atac.U5 tri-snRNP to the spliceosome [9]. Rearrangements in RNA-RNA interactions [9], [14], [15] and changes in protein composition [16] result in the formation of the catalytically active spliceosome, in which the U6atac and U12 snRNAs are base paired together and to 5′ss and BPS, respectively, to bring the catalytically important parts of the intron together for the first step of splicing [9], [17].

The U12-type spliceosome is believed to be essential in all organisms that have it, yet the relevance of maintaining two separate spliceosomal systems has remained elusive. Indispensable role at the whole organism level has been shown in *Drosophila* in which homozygous P-element insertions into the U12 and U6atac snRNA genes were found lethal at early embryogenesis and at the 3rd larval instar stage, respectively [18]. Similarly, the knockdown of specific protein components in mammalian cells arrests cell proliferation [11], [19]. A recent study reported that the *in vivo* splicing of U12-type introns is at least 2-fold slower than U2-type introns [20]. Consistently, increased levels of unspliced U12-type introns can be detected in the steady-state pool of cellular total RNA in both insect and vertebrate cells [21], [22]. The lower efficiency of U12-type splicing has been suggested to lead to nuclear retention and subsequent degradation of the transcripts thus providing rate-limiting regulation to a subset of genes and pathways [21], [23]. A single study suggested that U12-type splicing may instead take place in cytoplasm [24], but this work was subsequently challenged by several studies that showed that U12-dependent spliceosome components are localized in the nucleus and interact with components of the U2-dependent spliceosome, and that the splicing of U12-type introns takes place in the nucleus and is in fact co-transcriptional [20], [25], [26], [27].

We set out to investigate the impact of U12-dependent splicing on specific cellular pathways at the whole organism level. In mammals, genes containing U12-type introns have been assigned to a broad category of “information processing genes”, encoding for proteins operating at various stages of the gene expression pathway, and include members of several signaling pathways, cytoskeleton proteins and a large group of cellular ion channels [1], [28], [29]. In contrast to mammals that have more than 600 genes containing U12-type introns [4], [30], the *Drosophila* genome has only about 20 genes containing a putative U12-type intron [30], [31], [32]. Even though the number is more than an order of magnitude smaller than in mammalian genomes, most of the few *Drosophila* U12-type introns are conserved between mammals and flies [4], [30], and show a similar preference to information processing genes as their mammalian counterparts [1], [2], [31].

Here we used a *Drosophila* line with a P-element inserted into the U6atac snRNA gene and analyzed the genome-wide effects of the mutation on splicing and gene expression. Even though previous studies have shown that the homozygous U6atac mutation leads to at least a partial loss of splicing of U12-type introns [18], we found only relatively mild detrimental effects on the splicing of most genes containing U12-type introns, with significant levels of fully spliced mRNAs. Only two U12-type intron-containing genes were significantly downregulated in the flies carrying homozygous U6atac mutation. In contrast, our microarray analyses documented progressive perturbations in the expression of a large number of genes that do not carry U12-type introns, suggesting that defective U12-type intron splicing leads to significant downstream effects on genes that do not contain U12-type introns. Furthermore, our results suggest that the downstream effects may initiate from a single U12-type intron containing gene, a *Drosophila* homolog to the mitochondrial chaperone prohibitin, the expression of which is the most severely inhibited of all U12-type intron-containing genes.

## Results

### P-element insertion to U6atac gene leads to larval death

**Figure 1:**
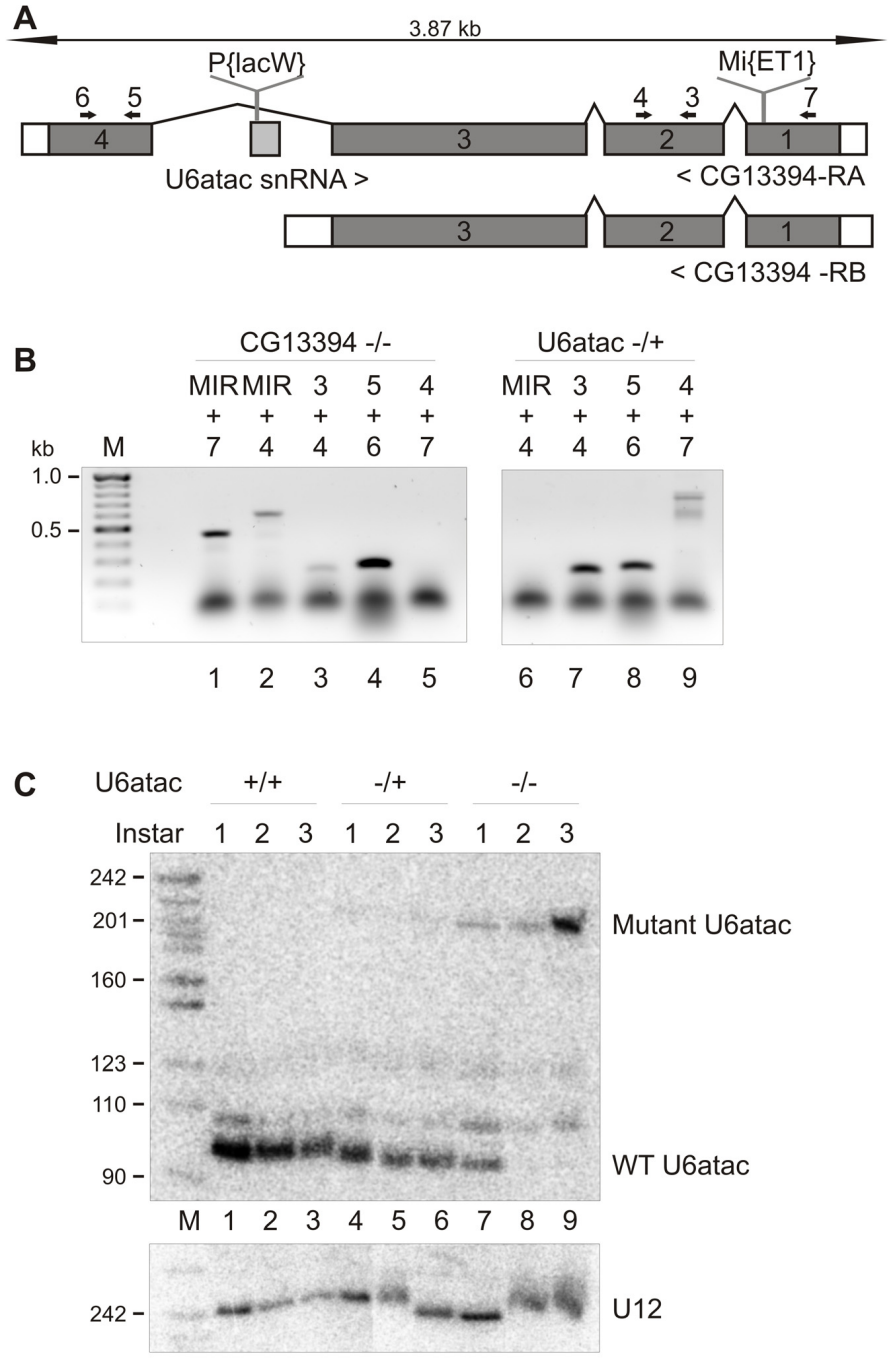
Description of U6atac and CG13394 mutations. **A.** Schematic picture of U6atac and CG13394 genes. Transposon insertion sites in the U6atac mutant (P{*lacW*}) and MB08402 line (*Mi{ET1*}) are indicated. Black arrows indicate the location of primers in CG13394 sequence. **B.** PCR amplification of cDNA sequences from MB08402 line (lanes 1–5) and U6atac −/+ line (6–9). Primer locations are indicated in A. MIR; primer oriented outwards from the inverted repeat sequence of the *Mi{ET1*} construct. **C.** Northern blot showing the expression of wt and mutant U6atac snRNA. Total RNA from wt (w^1118^; lanes 1–3), U6atac −/+ (lanes 4–6) and U6atac −/− (lanes 7–9) larvae in the three larval stages as indicated above was analyzed on a denaturing PAGE. The blot was probed also for the U12 snRNA as a loading control.

We used the *Drosophila* line *l(2)k01105* with a defect in the U12-dependent spliceosome to investigate the organismal level effects of U12-type intron splicing. This line contains a P{*lacW*} element inserted in the U6atac gene at position 70 (Fig. 1A), which leads to defects in the splicing of U12-type introns and larval lethality at the 3rd instar stage as described by Otake *et al.* [18]. Recent *Drosophila* genome maps indicate that the U6atac gene is located within an alternative intron of the protein-coding gene CG13394 (Fig. 1A). We found that the P-element insertion also leads to a loss of transcripts that contain the alternative exon 4 (not shown). To ask if any of the phenotypic effects of the P-element insertion are due to changes in CG13394 expression, we investigated the fly line MB08402 containing a putative null allele of CG13394 as a consequence of a *Mi{ET1}* transposon insertion in the first exon. Homozygous MB08402 flies were viable and fertile and did not display any apparent phenotypic abnormality. RT-PCR analysis using primers specific to *Mi{ET1}* transposon and CG13394 gene followed by sequencing confirmed the identity of chimeric transcripts containing both transposon- and CG13394-specific sequences (Fig. 1B, lanes 1, 2). Thus, while the insertion disrupts the reading frame of CG13394, it does not destabilize the chimeric mRNA. We conclude that the loss of a splicing isoform from CG13394 has a negligible effect on *l(2)k01105* phenotype, indicating that a defect in U12-type intron splicing by P-element insertion to U6atac leads to larval lethality.

We also confirmed the presence of chimeric U6atac snRNAs in the developing larvae. In the first instar larvae, maternally contributed wt U6atac is still present (Fig. 1C, lane 7), but by the third instar, wt U6atac disappears from homozygous mutant larvae and only the longer chimeric version is detected (Fig. 1C, lane 9) as described earlier [18]. In contrast, heterozygous larvae appear normal and exhibit no splicing defects compared to wt larvae (data not shown). Additionally, in heterozygous larvae, the levels of chimeric U6atac snRNA are low (Fig. 1C, lanes 4–6), possibly because of decreased stability, as suggested earlier [18]. Therefore, we used heterozygotes as controls in our subsequent experiments to minimize any variation due to environment or genetic background.

### Gene expression profiling

We investigated the effects of the U6atac mutation on global gene expression using custom microarrays. The array contained probes for most exons in the *Drosophila* genome annotated in the Ensembl database, except for few very short or low-complexity exons. Additionally, exon-exon junction probes were designed for genes containing U12-type introns, but these were ignored in the subsequent analysis after quality checks indicated that a subset of these long probes may bind even if the intron is present in the mRNA. Thus we considered them too unreliable for the quantification of spliced vs. unspliced products (not shown). Array experiments were conducted using total RNA samples from pools of first, second, and third instar larvae of U6atac −/− and U6atac −/+ lines.

Additionally, we used total RNA from 3rd instar larvae from the MB08402 line to identify genes potentially affected by mutations of the host gene (CG13394) of the U6atac snRNA. However, we did not detect any statistically significant changes in gene expression in the homozygous MB08402 larvae using exon microarray with cutoff p<0.01 (data not shown). This suggests that the effects detected in the U6atac mutant line *l(2)k01105* are caused by the disruption of U6atac snRNA and not by the loss of the last alternative exon of CG13394. This result is consistent with the observation that the homozygous MB08402 flies do not show any apparent changes in their viability or other phenotypic characteristics.

**Figure 2:**
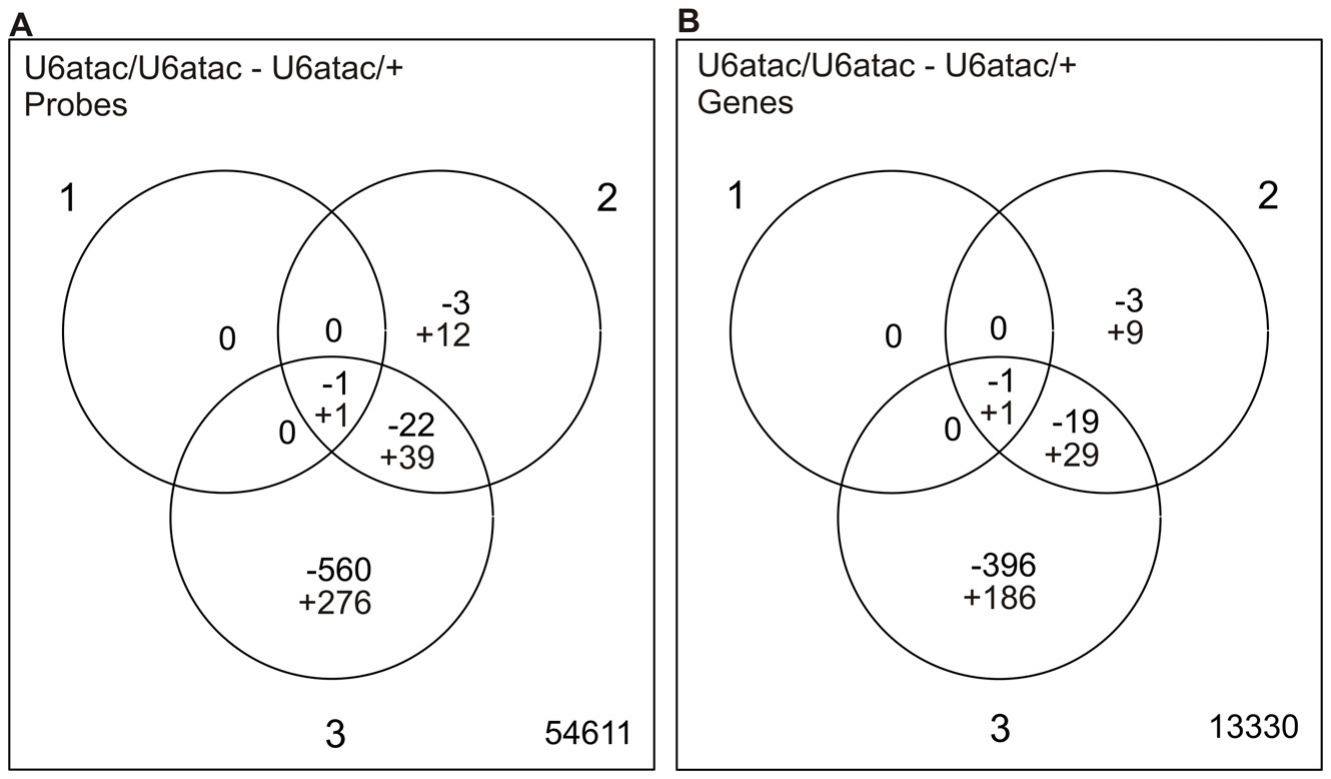
Venn diagrams depicting the microarray results. The numbers of significantly changed probes (**A**) and genes (**B**) at p<0.01 were plotted in the three larval stages. The positive numbers indicate upregulated and the negative downregulated probes or genes. The number of nonsignificant probes or genes is shown in the lower right corner of each panel.

In contrast, the homozygous U6atac/U6atac line showed progressive changes in gene expression during larval development. At the level of individual exon probes (Fig. 2A), only few statistically significant changes were observed at the 1st larval instar, but the number of statistically significant changes in gene expression was progressively escalated in 2nd and 3rd instar larvae. Importantly, the observed trend of gene expression changes, which mostly initiate at the 2nd instar when the maternally contributed wt U6atac snRNA runs out (Fig. 1C), is consistent with the scenario in which the loss of wt U6atac snRNA indeed triggers the observed changes in gene expression. Further support to this scenario is the observation that most of the detected gene expression changes are additive with respect to the larval developmental stage so that changes in the earlier developmental stages are observed also in the later stages (Fig. 2A). A similar pattern was also observed with a gene-level analysis (Fig. 2B). The majority of significant genes (416 out of total 632 genes) are downregulated in the U6atac −/− larvae at the 3rd instar. In contrast, at the 2nd instar, the majority of genes were upregulated (39 up, 23 down).

### U12-type intron-containing genes

**Figure 3:**
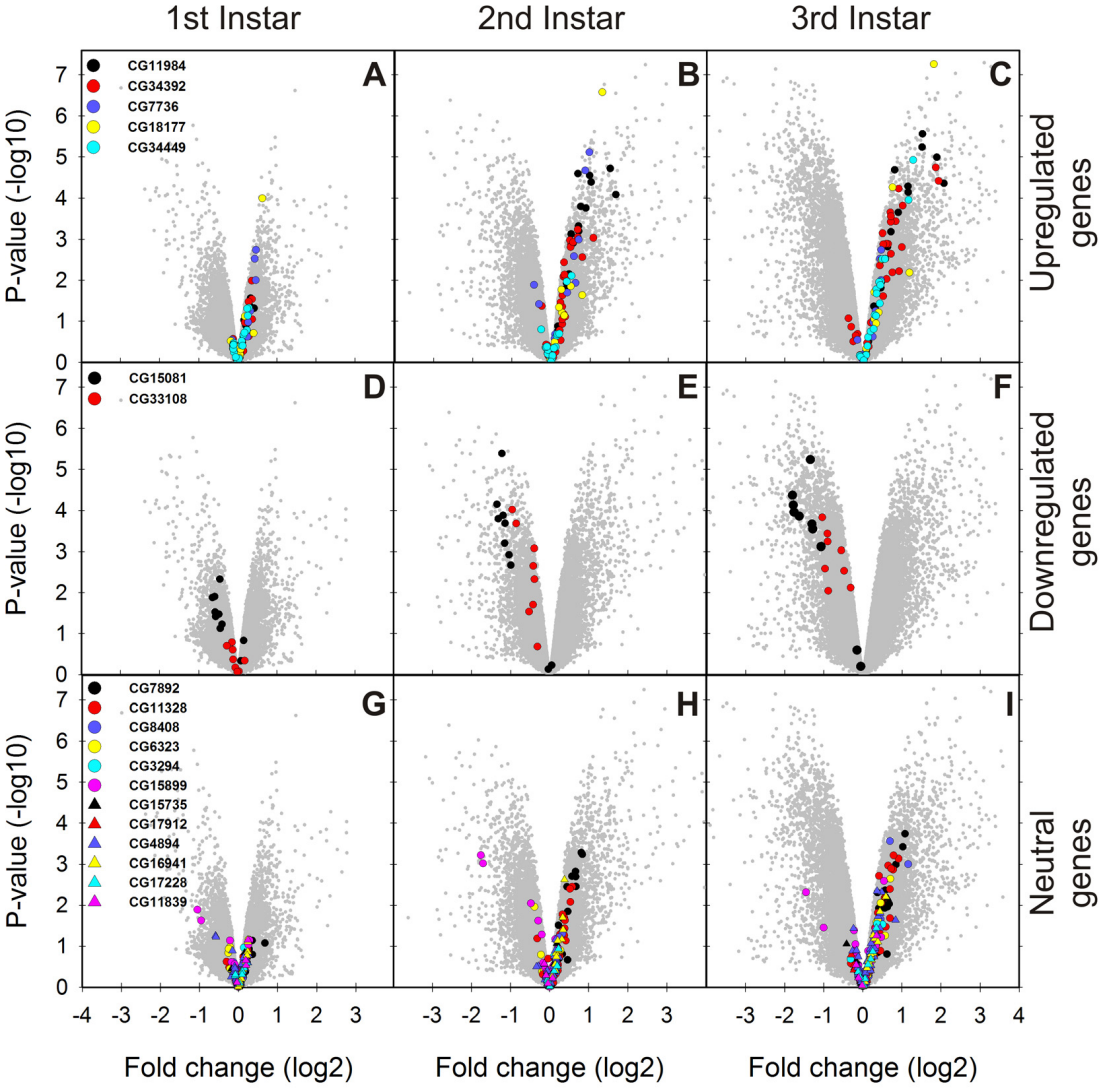
Volcano plots representing the probes for U12-type intron-containing genes. Fold change (x axis) was plotted against p value (y axis) in each larval instar. **A**–**C**. Upregulated probes. **D**–**F**. Downregulated probes. **G**–**I**. Nonsignificant probes.

Our expectation was that the mutation in the U6atac snRNA would primarily affect genes that contain U12-type introns as suggested earlier based on the analysis of a subset of *Drosophila* transcripts [18]. Quite surprisingly, our microarray results revealed only relatively mild effects on most of the 19 genes containing putative U12-type introns (Table 1). The results for genes containing U12-type introns are illustrated by probe-level volcano plots in which the expression levels and the statistical significance are plotted separately for the three differently behaving groups (upregulated, downregulated, and neutral) in each of the three larval stages. In the first group we observed clear effects of upregulation that became visible at the 2nd instar and was further enhanced in 3rd instar larvae (Fig. 3A–C). The strongest effects were seen with the two downregulated genes. The CG15081/l(2)03709 probes showed mild downregulation already at the 1st instar and the effect was exacerbated at subsequent stages (Fig. 3D–F). In contrast, the second downregulated gene, CG33108, was first affected at the 2nd instar, but was similarly further downregulated at the 3rd instar. In the unaffected U12-type intron group, most probes were slightly upregulated, but did not typically display any clear directional effect during larval development as seen in the other two groups (Fig. 3G–I). The two exceptions were CG7892/Nmo and CG11328/Nhe, which both showed moderate upregulation in 3rd instar larvae, but fell short of the statistical limit set in this study. The direction of the response with any of the genes containing a U12-type intron did not correlate with the properties of the gene or U12-type intron, e. g. position of the intron within the gene, U12-type intron subtype (GT-AG or AT-AC), or expression level.

**Table 1:** Probe-wise changes in U12-type intron genes.

Symbol	Flybase ID	Name	Human ortholog^1^	Unique probes	1st instar		2nd instar		3rd instar		Mutation phenotype^2^
					UP	DOWN	UP	DOWN	UP	DOWN	
CG11984	FBgn0037655		KCMF1	9	0	0	0	0	4	0	viable
CG34392	FBgn0085421	Epac	RAPGEF3	18	0	0	0	0	2	0	viable
CG7736	FBgn0037084	Syx6	STX6	5	0	0	1	0	2	0	lethal
CG18177	FBgn0036039		NAT15	5	0	0	1	0	2	0	viable
CG34449	FBgn0085478		ZDHHC8	10	0	0	0	0	1	0	viable
CG15081	FBgn0010551	l(2)03709	PHB2	5	0	0	0	1	0	3	lethal
CG33108	FBgn0053108		C19orf54	4	0	0	0	0	0	1	viable
CG7892	FBgn0011817	Nmo	NLK	8	0	0	0	0	0	0	lethal
CG11328	FBgn0028703	Nhe3	SLC9A7	15	0	0	0	0	0	0	viable
CG8408	FBgn0030850		TMEM41B	4	0	0	0	0	0	0	viable
CG6323	FBgn0039465	Tsp97E	TSPAN13	5	0	0	0	0	0	0	viable
CG3294	FBgn0031628		ZRSR2	3	0	0	0	0	0	0	viable
CG15899	FBgn0029846	Ca-alpha1T		23	0	0	0	0	0	0	viable
CG15735	FBgn0030364		LSM12	5	0	0	0	0	0	0	viable
CG4894	FBgn0001991	Ca-alpha1D	CACNA1D	31	0	0	0	0	0	0	lethal
CG16941	FBgn0038464	SF3a1	SF3A1	7	0	0	0	0	0	0	viable
CG17228	FBgn0004595	Pros	PROX1	4	0	0	0	0	0	0	lethal
CG11839	FBgn0039271		ZNF830	2	0	0	0	0	0	0	viable
CG17912	FBgn0032600		ZNF207	7	0	0	0	0	0	0	lethal

1 Ensembl release 58.

2 Phenotypes inferred from Flybase FB2010_04.

**Figure 4:**
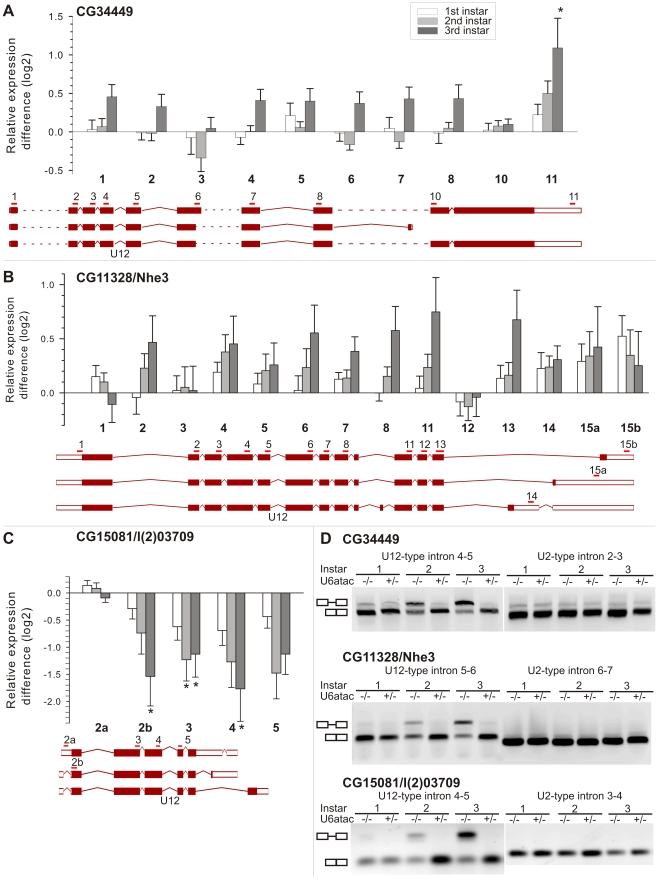
Expression changes in three representative U12-type intron-containing genes. **A**–**C.** Probe-wise microarray results for three genes representing upregulated (A), nonsignificant (B) and downregulated (C) categories. Error bars indicate standard deviation. Significant changes at p<0.01 are marked with ‘*’. Schematic drawings of the most common splicing isoforms that indicate the positions of individual probes (horizontal bars and probe numbers above the exons) and U12-type introns are shown below each bar chart. Filled rectangles indicate exons of the coding sequence and open rectangles UTR regions. Introns are indicated as lines; the dotted lines indicate large introns that have been truncated in the schematic picture. **D**. Validation of microarray results by RT-PCR. Primers were located in exons at either side of the intron indicated.

We next investigated if the U6atac mutation would cause systematic effects on the splicing of U12-type intron-containing genes by plotting the exon array data of those genes and comparing this to the structures of individual genes. Besides a slight bias for more pronounced fold-changes for 3′-end probes, which is probably introduced during cDNA synthesis or amplification steps, we did not detect any consistent general changes that would have indicated unusual aberrant splicing patterns near the U12-type introns. A representative set of individual genes belonging to each of the three classes of U12-type intron-containing genes (upregulated, neutral, and downregulated) are presented in Figs. 4 and S1. From this data we noticed that both the upregulated and neutral genes on the array shared a similar trend in which the individual exon signals are typically progressively upregulated at the 2nd and 3rd instars. For example, the upregulated CG34449 gene (Fig. 4A) showed only one statistically significantly upregulated exon at the 3′ end, but most of the other probes were also upregulated in 2nd and 3rd instar larvae without reaching the statistical cut-off set in the study. The neutral gene CG11328/Nhe (Fig. 4B) did not show any statistically significant changes at the probe level even though all the probes were slightly upregulated (or neutral, as probe 12). In contrast, most of the probes for the downregulated gene CG15081/l(2)03709 were significantly downregulated in the homozygous U6atac mutant larvae, with several probes reaching the statistical cut-off of the study (Fig. 4C). The exception was probe 2a, which specifies an mRNA species containing a retained intron in the 5′UTR. This probe displayed a low signal in our array data indicating that such mRNAs have low expression levels.

To further investigate the effect of the U6atac mutation on U12-type intron splicing, we analyzed genes from each category by RT-PCR with primers flanking a short U12- or U2-type intron to detect both unspliced and spliced mRNAs in the same reaction. Consistent with the splicing defect reported earlier [18], we detected significantly elevated levels of unspliced U12-type introns from genes belonging to each of the three categories in the homozygous U6atac mutant larvae (Fig. 4D). Additionally, we also detected substantial levels of spliced U12-type introns (Fig. 4D), suggesting that the U6atac mutation does not completely abolish the activity of the U12-dependent spliceosome. Therefore, the fully processed mRNA levels for the U12-type intron-containing genes are somewhat lower than estimated from the array data, which does not distinguish partially processed and fully processed mRNAs. The outcome is, as judged from the ratio of spliced vs. unspliced U12-type introns in Figure 4D, that the functional mRNA levels are either near the wt level or somewhat reduced for most of the upregulated or neutral genes and severely reduced for the two downregulated genes.

### Enrichment of genes encoding for metabolic functions on U6atac −/− larvae

**Figure 5:**
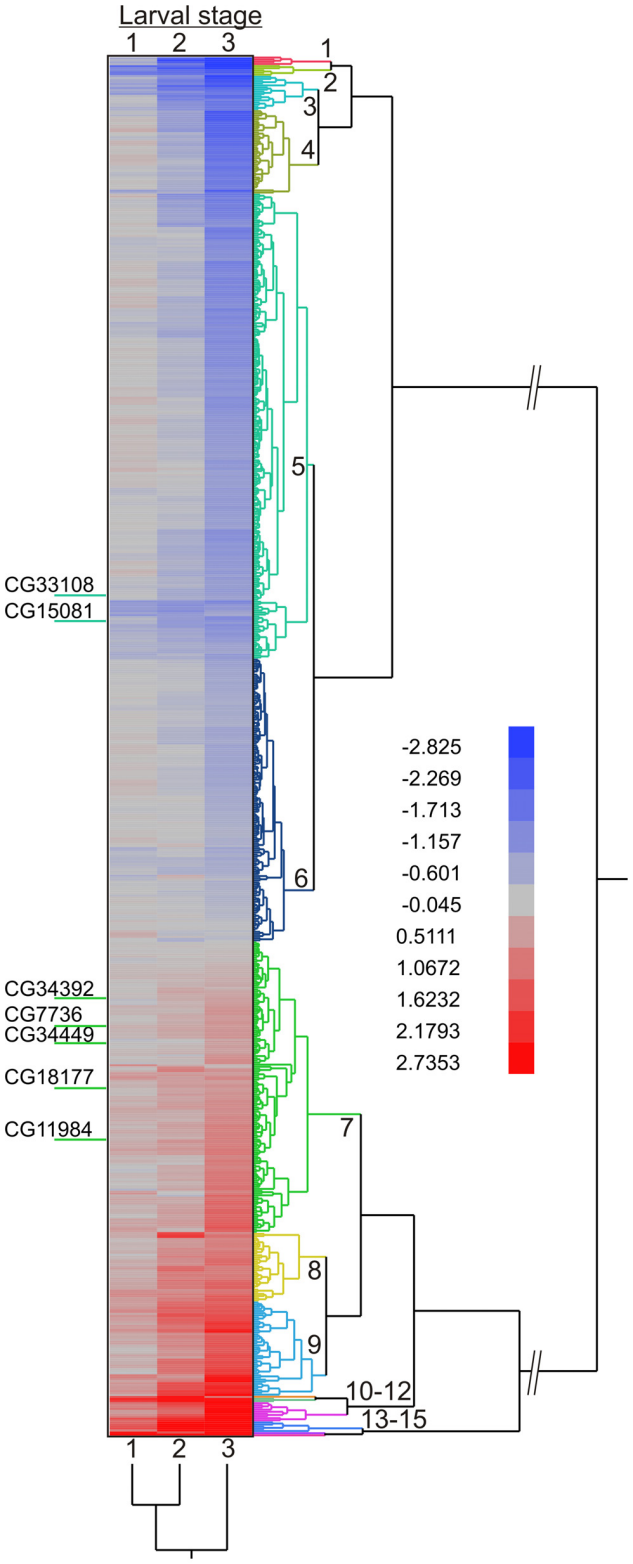
Hierarchical clustering of the significant genes in the three larval stages. Clustering was done in JMP/Genomics 7.1/3.1 (SAS) using two-way clustering, with complete clustering method. The input data was the mean expression difference (probe average) between U6atac −/− and −/+ larvae for the genes that contained one or more statistically significant probes. The U12-type intron-containing genes are highlighted in the heatmap. The genes containing U12-type introns have been indicated on the left. The cluster numbers on the right relate to the supplementary Table S1.

Most of the genes affected in the 2nd and 3rd instars did not contain U12-type introns (Fig. 2). Presumably they are situated downstream in the pathways that are disturbed by the splicing defect in the (few) primary genes containing U12-type introns. We next sought to understand which of the cellular pathways are disturbed in the U6atac −/− larvae as this would also help to identify those U12-type genes that are primarily responsible of larval lethality. First we used clustering analysis on the array data to ask the association of U12-type intron-containing genes with the other genes displaying statistically significant changes in our array experiment. A heatmap showing expression differences in each larval stage is shown in Figure 5. We found that the five upregulated U12-type intron-containing genes are clustered in a group of mildly upregulated genes (cluster 7 in Figure 5), whereas the two downregulated genes are in the same slightly downregulated group (cluster 5). The ordered list of significantly changed genes was subjected to GO term analysis using DAVID functional annotation tool (Table S1) [33], [34], [35]. Enrichment analysis was done separately for each cluster in the heatmap, for all up- or downregulated clusters, and for all clusters combined. We found that the most highly upregulated clusters were enriched with genes involved in defense response (such as drosomycin-5 involved in fungal defense) and several groups of enzymes with detoxification-related transferase activities such as members of P450 family, UDP-glucuronosyl/UDP-glucosyltransferase and glutathione-S transferases (Table S1). In contrast, the most downregulated clusters were enriched with genes related to lipid, nucleotide, and amino acid metabolism. A further KEGG pathway analysis implicated several metabolic pathways, including amino acid degradation and fatty acid metabolism, as being affected (Table S1). We validated several of the most affected genes by qRT-PCR in the three larval stages and found a good correlation between the microarray and qRT-PCR results (Table 2).

**Table 2:** Validation of array results by quantitative RT-PCR.

Symbol	Flybase ID	Name	Instar	Array log_2_-fold change	qPCR log_2_-ratio^1^
CG10091	FBgn0038020	GstD9	1	0.7642	1.4359
			2	2.0693	3.6536
			3	3.0899	3.1499
CG10812	FBgn0035434	Dro5	1	2.7641	−3.4804
			2	4.0783	4.0391
			3	6.5669	10.1461
CG4486	FBgn0015039	Cyp9b2	1	1.2309	0.2940
			2	3.2867	4.7071
			3	2.7985	2.9747
CG8864	FBgn0028940	Cyp28a5	1	−1.6696	−4.2821
			2	−1.6160	−1.8039
			3	−2.2946	−1.5560
CG11455	FBgn0031228	Gh23780p	1	−1.0328	−0.1779
			2	−1.3075	−0.6010
			3	−1.5422	−0.2134

1 The results were calculated with Lightcycler software (Roche Diagnostics) and the control/mutant ratio was transformed into log_2_ space for comparison.

As the lethal phenotype in the U6atac −/− larvae is most likely mediated by a small number of U12-type intron-containing genes acting upstream, we analysed their possible links to metabolic pathways or lethal larval phenotype. Of all the U12-type intron-containing genes in *Drosophila*, six are known to have lethal mutation phenotypes (Table 1). Importantly, among those is CG15081/*l(2)03709*, the most significantly downregulated U12-type intron gene in U6atac −/− larvae, which encodes an ortholog to *prohibitin 2* (PHB2). Prohibitin is an essential mitochondrial protein conserved in eukaryotes from yeast to mammals [36] and functions as a holdase/unfoldase chaperone stabilizing membrane proteins [37]. As this U12-type intron-containing gene displayed the most severe effects in the U6atac mutant with strong downregulation at the 2nd and 3rd instars (Fig. 3E, F) we considered it to be the most likely candidate leading many of the phenotypic changes observed in the U6atac −/− larvae.

**Figure 6:**
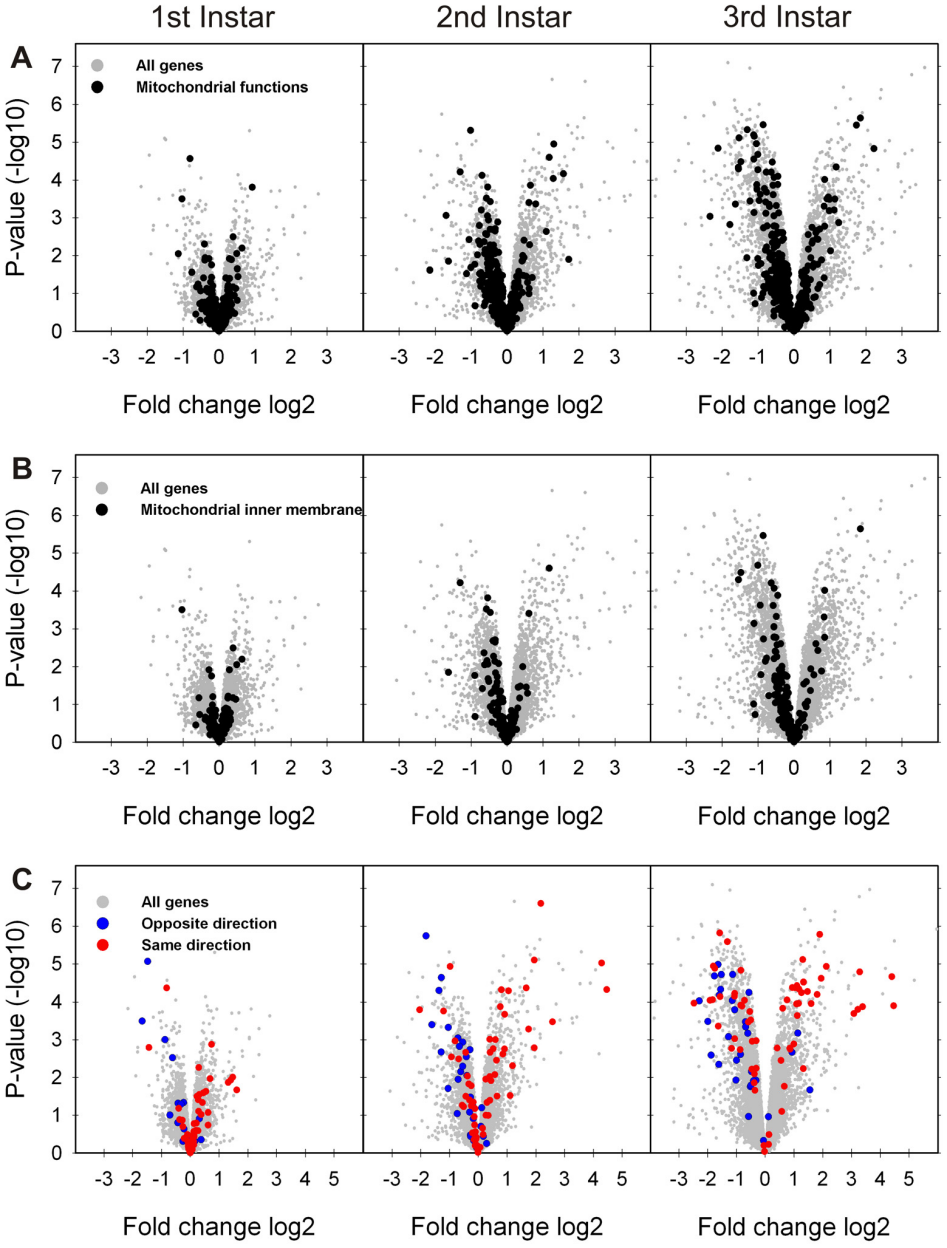
The effect of U6atac mutation on the expression of mitochondrial genes. **A.** Volcano plots showing the effect of the U6atac mutation on all nuclear-encoded mitochondrial genes in the three larval stages. **B.** Volcano plots showing the effect of the U6atac mutation on the expression of genes encoding for mitochondrial components related to the inner membrane. **C.** Comparison of the expression profiles of U6atac −/− and *tko* mutants. Microarray results from 3rd instar U6atac −/− larvae were compared with *tko* adults [38]. The red color indicates significant genes in the present study that show unidirectional change in both U6atac −/− and *tko* lines (either up- or downregulated). The blue color indicates significant genes in the present study that show the opposite direction of change between the U6atac −/− and *tko* lines.

To find out if the expression of genes encoding for mitochondrial components was affected in the U6atac larvae, we further analyzed the effect of the U6atac mutation on the expression of nuclear-encoded genes linked to mitochondrial functions. As shown in Figure 6, we detected widespread changes in the expression of genes related to mitochondrial functions that were exacerbated towards the 3rd larval stage. Even though there were both up- and downregulation in the mutant flies, the downregulated genes formed the most prominent group (Fig. 6A). Among genes linked to mitochondrial inner membrane, the bias towards negative expression change was even stronger (Fig. 6B). Finally, we compared our data to a recent microarray study in which a *Drosophila* line with a mutation in the *technical knockout* (*tko*) gene that encodes the mitoribosomal protein S12 was analyzed using Affymetrics platform [38]. We did this comparison because *prohibitin* mutations have not been analysed in genome-wide fashion in *Drosophila*. In the *tko* mutant line, 970 genes were identified as significantly altered in males and/or females. We found that ∼15% of the significant genes (95 out of 644 genes) in the U6atac −/− line were shared with the *tko* line. Assuming that both experiments shared all the unique FlyBase genes present on our array (13764 genes), the number of shared genes was 2.1-fold higher than expected by chance (p<2.6*10^−12^, hypergeometric test). Furthermore, 67% of these displayed the same direction of change in both studies (28 up- and 36 down-regulated, p<6.6*10^−5^ in Fisher's exact test; see Fig. 6C and Table S2). The similarities were remarkable because in the Fernández-Ayala et al. [38] study the flies survived until adulthood and were sampled at this stage, compared to the 3rd instar larvae used in our study. We conclude that the phenotypic and gene expression level effects observed with U6atac −/− line are most likely initiated with a defect in the splicing of the U12-type intron in *prohibitin* gene. However, the splicing defects in the other genes containing U12-type introns probably exacerbate this and further contribute to the larval lethality.

## Discussion

The *Drosophila* line with a P-element insertion in the U6atac gene is unique as it allows investigations on U12-type intron splicing defects at the organismal scale. An earlier study reported that this mutation is lethal at the 3rd larval stage [18], but the information on gene expression changes was limited to few genes containing U12-type introns. Here we used exon microarrays to investigate the genome-wide effects of the U6atac mutation on larval gene expression. We found that this mutation leads to a large number of cumulative changes in gene expression, particularly in 2nd and 3rd instar larvae (Figs. 2, 5). Surprisingly, we found that the exon signals of most genes containing U12-type introns were not significantly affected by the U6atac mutation. Of the nineteen documented genes containing U12-type introns only two were downregulated and five were upregulated at the exon probe level. However, in each case RT-PCR analysis detected both unspliced and spliced U12-type introns. The residual U12-type splicing activity is most likely due to a low level of activity of the chimeric U6atac snRNA containing both U6atac and P-element sequences. In addition to the observed changes in U12-type intron-containing genes, a large number of genes that do not contain U12-type introns were either up- or downregulated. Enrichment analysis indicated that genes related to defense response and detoxification of xenotoxic compounds were the most significant upregulated gene groups. Conversely, genes related to various cellular metabolic pathways were enriched among the downregulated genes.

The relatively weak effect of the U6atac mutation on the mRNA levels of the genes containing U12-type introns was unexpected since the U6atac snRNA is a central component of the U12-dependent spliceosome and is involved in the splicing catalysis [9], [14], [17], [39], similarly as the U6 snRNA in the U2-dependent spliceosome [40], [41]. A more detailed analysis of a subset of U12-type intron-containing genes that were either upregulated or not affected by the U6atac mutation revealed a substantial level of splicing activity of U12-type introns, but also accumulation of mRNA species containing unspliced U12-type introns (Fig. 4D). Together these results suggest that there are two mRNA populations derived from U12-type intron-containing genes: fully spliced mRNAs that are presumably efficiently exported to the cytoplasm, and mRNAs that contain unspliced U12-type introns that are most likely retained in the nucleus and possibly subject to nuclear degradation pathways. RT-PCR analysis indicated that in the U6atac −/− larvae the levels of spliced U12-type introns are approximately 30–50% of the levels found in heterozygotes (Fig. 4D) or wt larvae (not shown). Thus our results reinforce the earlier suggestion that the mutated U6atac snRNA may have retained partial catalytic activity [18]. Alternatively, trace amounts of maternally contributed wt U6atac snRNA that were not visible in our Northern blot analysis might still be present in 3rd instar larvae.

Paradoxically, the mRNA-level response to the U6atac mutation among the individual U12-type intron genes was highly variable, and most genes containing U12-type introns did not display any changes at the exon probe level (which measures both spliced and unspliced mRNAs), while a subset showed either up- or downregulation. This result is possibly linked to nuclear retention and/or degradation of the unspliced mRNAs, which may vary between the individual genes similarly as has been described previously in yeast microarray studies of the U2-dependent spliceosome [42], [43]. Similarly to the yeast studies, we were not able to identify any common denominators, such as expression level, splice site strength, or U12-type intron subtype, from the small group of *Drosophila* U12-type intron-containing genes that would have explained the observed gene-specific differences.

Many of the upregulated or neutral genes containing U12-type introns displayed substantial amounts of fully spliced mRNA and therefore it was questionable whether their mRNA levels would be low enough to contribute to the larval lethality. In contrast, two genes, CG15081/*l(2)03709* and CG33108 showed strong downregulation, suggesting that their transcripts may become unstable upon deficient processing. We hypothesized that one or both of these genes may play a central role in the larval death, since they are significantly affected already at the 1st or 2nd larval stage at the same time when the maternally contributed wt U6atac snRNA is depleted in the U6atac −/− larvae (Fig. 1, 3D–F). Our enrichment analyses of the genes significantly affected by the U6atac mutation revealed that many metabolic pathways were affected. Earlier reports have shown that genes linked to metabolic functions are mostly devoid of U12-type introns that are instead concentrated in so-called “information processing genes” [1], [4], [28]. Even though the number of *Drosophila* genes containing U12-type introns has been significantly reduced during dipteran evolution, a similar enrichment can be observed in this organism (Table 1, [32]). Only one gene of a known function, CG15081/*l(2)03709*, can be directly linked to metabolic pathways. CG15081/*l(2)03709*, the fly homologue of *prohibitin 2,* is the most severely downregulated gene in U6atac −/− larvae that contains a U12-type intron. The prohibitin complex consists of two subunits, PHB1 and PHB2, which have multiple roles in the cell. The most prominent function of the PHB complex is in the mitochondrial inner membrane, where it is believed to function as a holdase/unfoldase chaperone stabilizing membrane proteins [37]. It also has roles in the maintenance of mitochondrial morphology and the degradation of mitochondrial membrane proteins [44], [45]. Disruption of the PHB complex causes premature ageing accompanied with accumulation of mitochondrial defects and shortening of replicative lifespan in yeast [46], larval death in *Drosophila* [47], and more complex and severe phenotypes in other multicellular organisms, including animals and plants [48], [49]. Our finding that genes encoding for mitochondrial proteins, in particular those related to the inner membrane, are affected in the U6atac −/− larvae suggests a widespread mitochondrial dysfunction (Fig. 6A,B) and supports the important role of *prohibitin* in larval death. More importantly, a comparison of our data with microarray data from the *tko* fly line with a mutation in the mitochondrial protein S12 [38] revealed that a subset (∼15%) of genes affected in the U6atac mutant are also affected in the *tko* line despite the differences in developmental stage (adult vs. larvae) or different microarray platform used. Remarkably, 67% of the affected genes showed a similar expression change between the two lines (Fig. 6C, Table S2). The shared genes include detoxification-related transferases, cytochrome P450 genes and enzymes with functions in nucleotide, amino acid and fatty acid metabolism, which all are among the most affected genes in our analyses. Strikingly, the metabolic genes detected in our study, particularly those related to nucleotide and amino acid metabolism, are upregulated after overexpression of MnSOD in *Drosophila* mitochondria, which extends the lifespan in *Drosophila* by decreasing the effect of oxidative stress in mitochondria and by modulating a variety of metabolic pathways through retrograde signaling of nuclear genes [50]. Similar signaling pathways may lead to the downregulation of metabolic genes in mutant U6atac flies as a consequence of prohibitin downregulation.

In summary, we conclude that in *Drosophila*, the deficiency in U12-type splicing caused by a P-element insertion to the U6atac gene leads to a relatively mild splicing defect of U12-type introns. In most cases, accumulation of pre-mRNAs containing unspliced U12-type introns, but also formation of fully spliced mRNAs, is observed. Additionally, the expression of genes related to several metabolic pathways is significantly downregulated while genes related to defense responses are markedly upregulated. While it is likely that the U6atac mutant phenotype results from the combined influence of insufficient expression of several U12-type intron-containing genes and their downstream effects, our data suggests that disruption of the prohibitin complex functions may play a central role.

## Methods

### Fly strains

The U6atac mutant line *l(2)k01105* [51] and the CG13394 mutant line MB08402 (stock BL26111) were obtained from Bloomington fly stock center, Indiana, USA. The flies were maintained on standard food at 25°C.

### RNA isolation

Larvae were homogenized with Ultra-Turrax tissue homogenizer (IKA-Werke, Staufen, Germany) in Trizol reagent (Invitrogen) followed by RNA isolation according to manufacturer's instructions except additional acid phenol (pH 5.0) and chloroform extractions were performed prior to precipitation. RNA samples were treated with the DNA-free kit (Ambion/Applied Biosystems) prior to use in RT-PCR or microarray experiments.

### RT-PCR

RNA samples were reverse transcribed with RevertAid M-MuLV (Fermentas) according to manufacturer's instructions using a d(T)_20_ primer. PCR was performed with 26–28 cycles of 94°C for 30 s, 62°C for 30 s, 72°C for 30 s. Products were analyzed on standard agarose or MetaPhor agarose (Lonza) gels with SYBR Green I (Invitrogen) staining and imaged on LAS-3000 (Fujifilm).

### Quantitative PCR

qPCR was performed with Lightcycler 480 real-time PCR system (Roche Diagnostics) in 10 µl reactions on 384-well plates using Lightcycler 480 SYBR Green I Master complemented with 5 pmol of primers and cDNA corresponding to 40 ng of total RNA used in reverse transcription. Three replicates for each reaction were included in the PCR runs. Results were analyzed with Lightcycler Software 1.5.0.39.

### Northern blotting

Total RNA was analyzed in denaturing polyacrylamide gels (PAGE) as described [22], probed with full-length probes labeled with Rediprime II (Amersham/GE Healthcare) and imaged using BAS-1500 phosphoimager (Fujifilm).

### Microarray design

A total of 69,975 *Drosophila melanogaster* exon sequences were retrieved from the Ensembl database (build BDGP5.4) via BioMart service [52]. The fasta-formatted exon sequences were imputed into Agilent eArray webservice and 64,558 60-mer probes were retrieved by using the default parameters (https://earray.chem.agilent.com/). The probes were further tested for possible partial cross hybridization and 56,163 probes were finally utilized for producing 105 K Agilent custom arrays. This set of probes covers all the annotated exons in *Drosophila* but excludes some very short or low-complexity exons. In addition, probes spanning the exon-exon junction were designed for each junction in genes containing a U12-type intron, but these probes were later excluded from the analysis due to unreliability. When analyzed independently, the probes allowed screening the expression of 56,163 exons; additionally, when grouped by gene, they could be used to measure the expression of 13,974 genes.

### Microarray experiments

1 µg of each RNA sample was labeled with Amino Allyl MessageAmp™ II aRNA Amplification Kit (Ambion/Applied Biosystems) according to instructions. In vitro transcription reaction was incubated for 8 hours. Cy3 or Cy5 dyes (Invitrogen) or ARES Labeling Kit with Alexa Fluor 488 (Invitrogen) was used to label 5 µg (Cy dyes) or 3.5 µg (Alexa dye) of resulting aminoallyl-modified RNA. 0.75 µg of each three samples per array were hybridized with Gene Expression Hybridization kit (Agilent). Replicate experiments were made with each three colors for each sample except U6atac −/+ third instar, for which only two replicates were made. For CG13394 experiments, no replicates were made.

### Microarray data analysis

The gpr files were imported into R 2.9.0 (cran.r-project.org/) and analyzed with the BioConductor (www.bioconductor.org) package Limma [53]. Data exploration and quality control were performed using the Limma package. The median foreground probe intensities were utilized for the analysis, without any background correction, as recommended by Zahurak et al. [54]. For the exon-wise analysis, the probe values from each channel were log_2_ transformed and normalized by the quantile method [55]. The data have been deposited in NCBI Gene Expression Omnibus database (accession number GSE24148). For the gene-wise analysis, the average expression values per gene were similarly processed.

Analysis of variance and moderated t-test were carried out for finding the differentially expressed exons and genes in each developmental stage, using the methods implemented into the Limma package. Furthermore, Fisher's exact test was used for finding biological themes over-represented in the differentially expressed gene lists with DAVID software using GO term (biological process, cellular component, molecular function), Interpro domain and KEGG pathway enrichment with default settings [35].

Statistical comparison of U6atac and *tko* mutant array data was done with hypergeometric test (http://elegans.uky.edu/MA/progs/overlap_stats.html). In the *tko* mutant, some genes were significantly expressed in only one sex. The comparison of the direction of expression changes between the two experiments was carried out using the expression data from male *tko* mutant flies, when available (only one of the genes shared between U6atac mutant and *tko* data changed in the opposite direction in *tko* females than males), using Fisher's exact test.

## Supporting Information

Figure S1 Expression changes in U12-type intron-containing genes. Plots depict probe-wise microarray results for upregulated (CG11984, CG34392, CG7736 and CG18177), downregulated (CG33108) and nonsignificant (CG7892, CG8408, CG6323, CG3294, CG15735, CG16941, CG17228, CG11839 and CG17912) U12 intron genes. Similar plots for CG34449, CG15081 and CG11328 are shown in Fig. 4. CG15899 and CG4894 were not plotted due to a large number of exons. Error bars indicate standard deviation. Significant changes at p<0.01 are marked with ‘*’. Schematic drawings of the most common splicing isoforms that indicate the positions of individual probes (horizontal bars and probe numbers above the exons) and U12-type introns are shown below each bar chart. Filled rectangles indicate exons of the coding sequence and open rectangles UTR regions. Introns are indicated as lines; the dotted lines indicate large introns that have been truncated in the schematic picture.
(0.25 MB PDF)

Table S1 Functional analysis of significantly changed genes. Significantly changed genes in 1st, 2nd or 3rd instar U6atac −/− larvae compared to control are ordered by hierarchical clustering (sheet 1). The coloring of the clusters corresponds with the heatmap shown in Fig. 5; for description of the clustering method, see legend to Fig. 5. Other sheets show an enrichment analysis with DAVID software (see Methods for details) for GO-terms (BP, biological process; CC, cellular component; MF, molecular function), protein domains (Interpro) and KEGG pathways, in different levels of the hierarchical cluster (all clusters combined, up- or downregulated clusters only and individual clusters separately). Results of the enrichment analysis are shown for each of the terms (sheets 2, 4, 6 & 8), and grouped by functional clustering method (sheets 3, 5, 7 & 9).
(1.57 MB XLS)

Table S2 Comparison of array data from U6atac and *tko* mutants. Array data from the U6atac mutant was compared to the *tko* mutant data based on Flybase identifiers and the 95 shared genes were grouped according to the direction of expression change. In the *tko* experiment, males and females were studied separately. Here, data from males was used for comparisons when available. Expression values are indicated as fold change.
(0.10 MB XLS)
