# Supplementary figures and images for: Gene Expression Profiling of U12-Type Spliceosome Mutant Drosophila Reveals Widespread Changes in Metabolic Pathways
Source: PLoS One. 2010 Oct 11;5(10):e13215. doi: 10.1371/journal.pone.0013215 (PMC2952598; doi:10.1371/journal.pone.0013215)

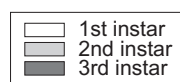

**CG11984**

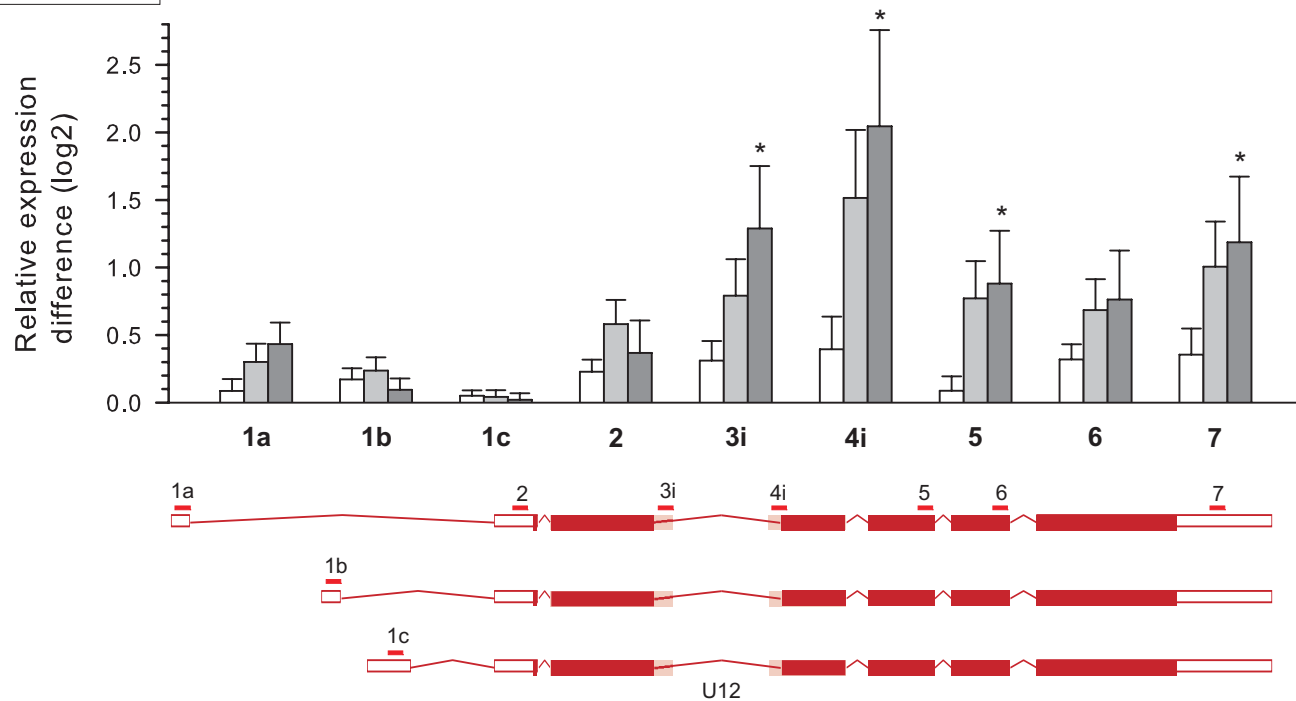

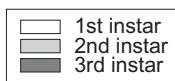

# CG34392 (Epac)

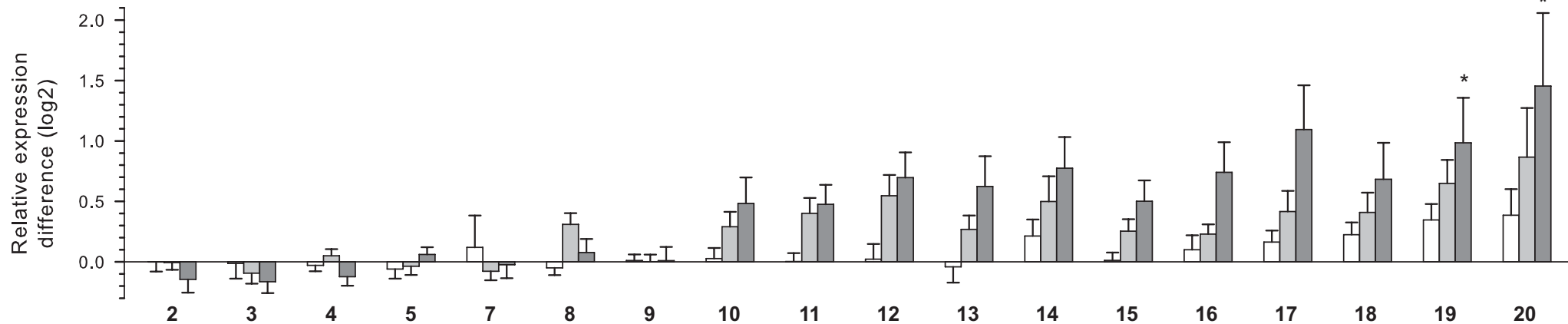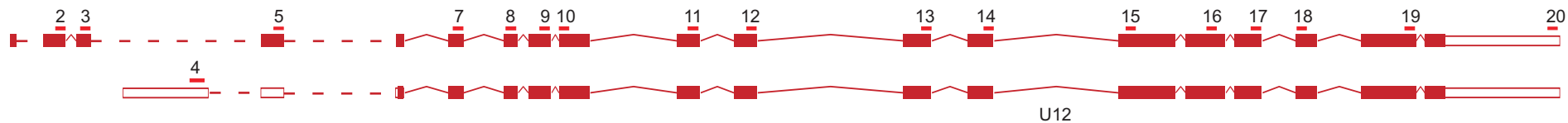

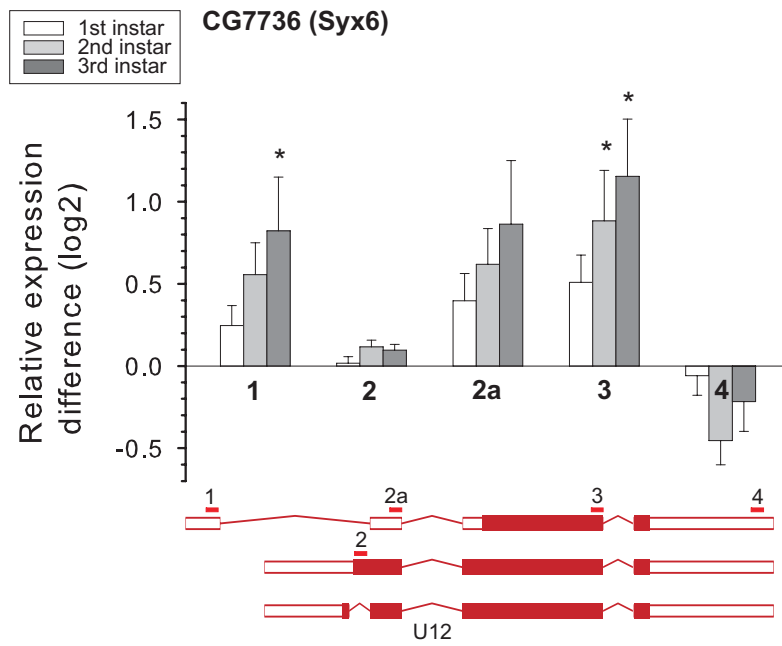

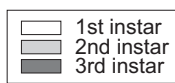

## CG18177

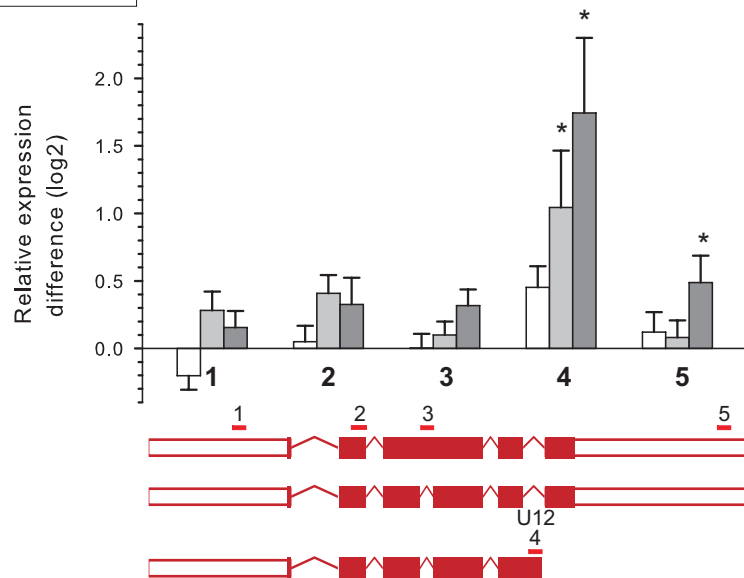

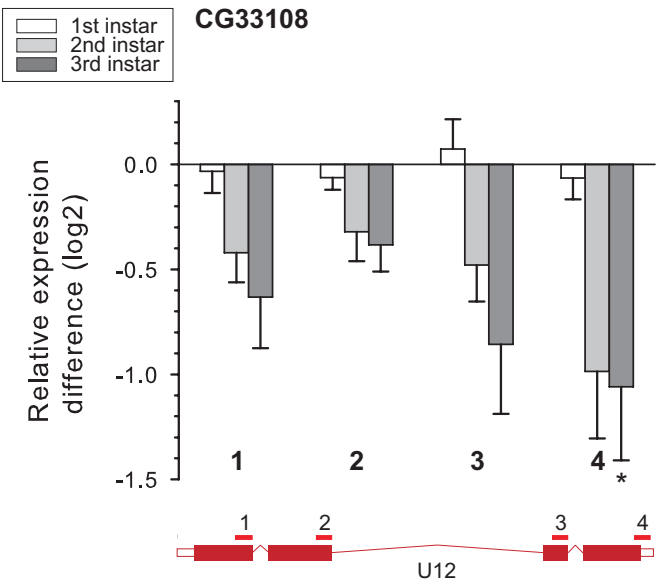

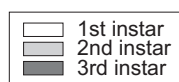

# CG7892 (Nmo)

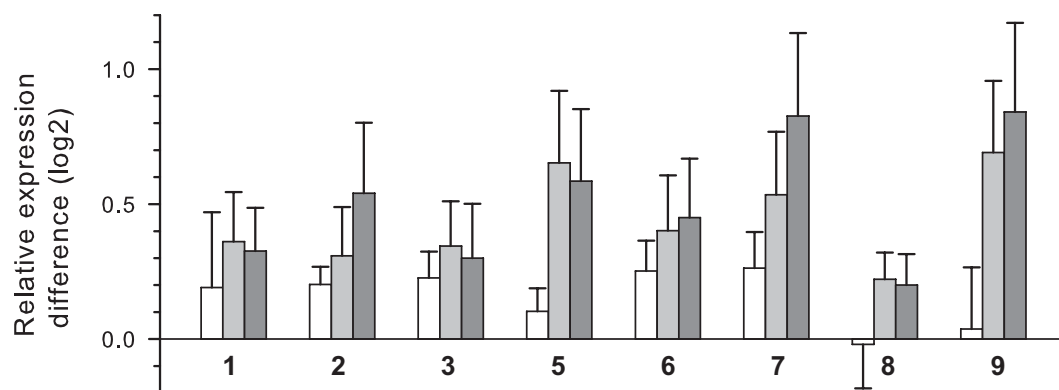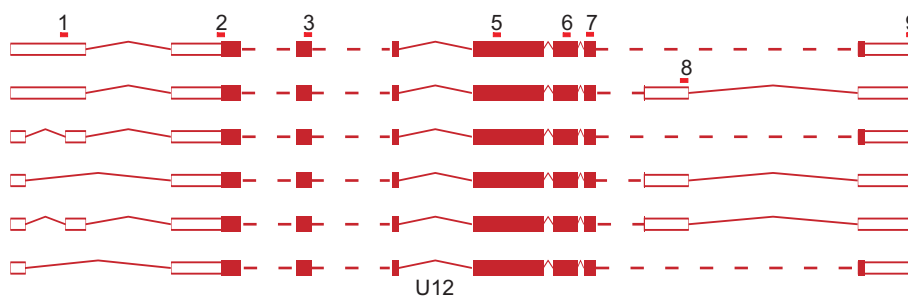

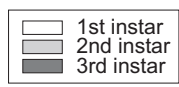

**CG8408**

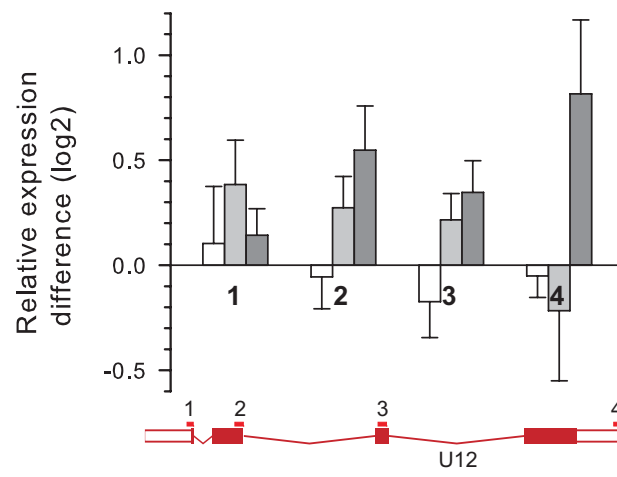

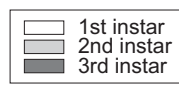

### CG6323 (Tsp97E)

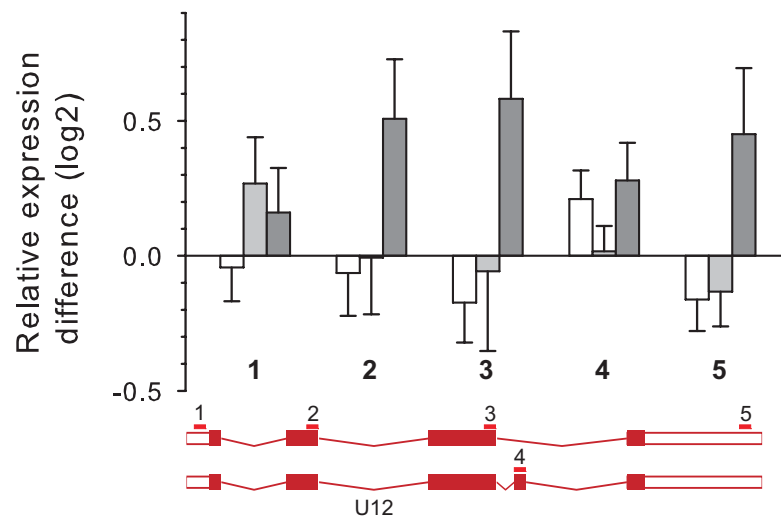

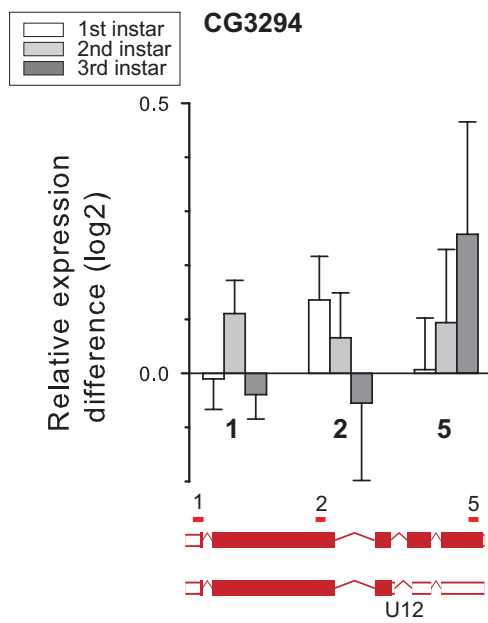

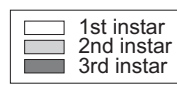

**CG15735**

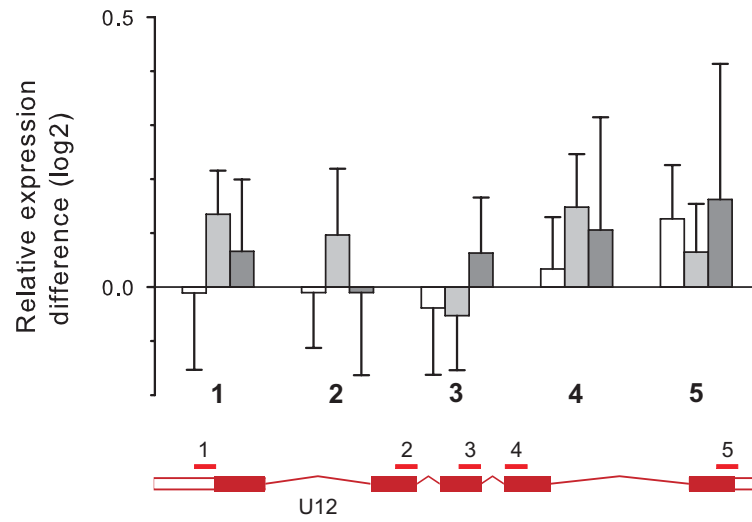

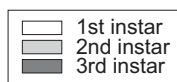

### CG16941 (SF3a1)

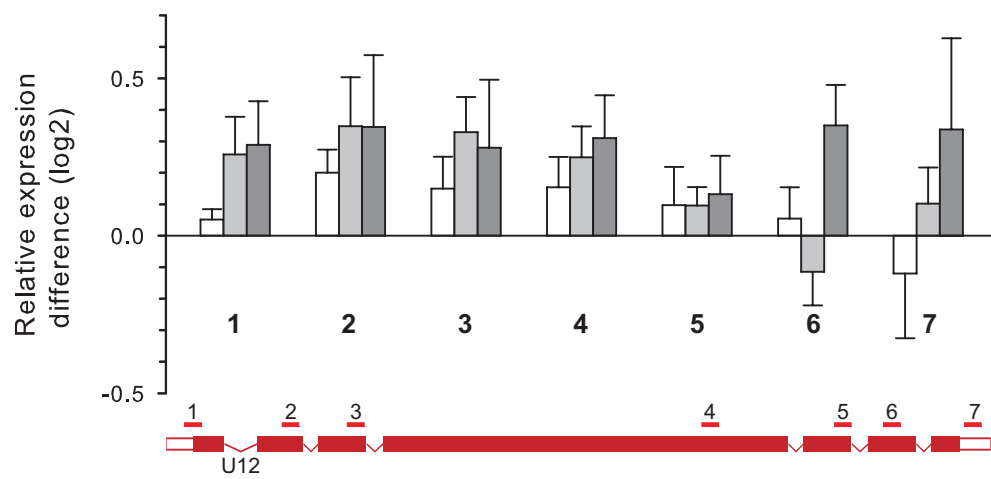

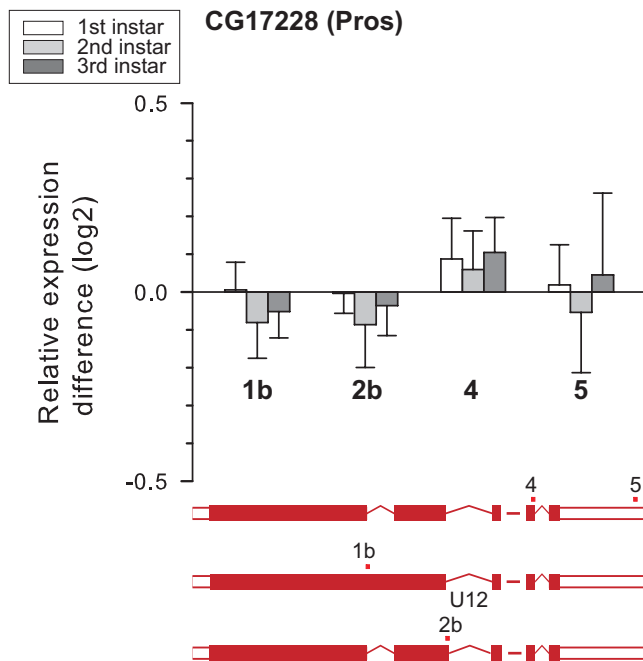

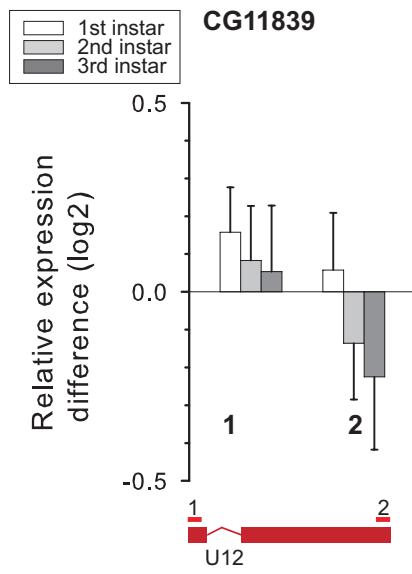

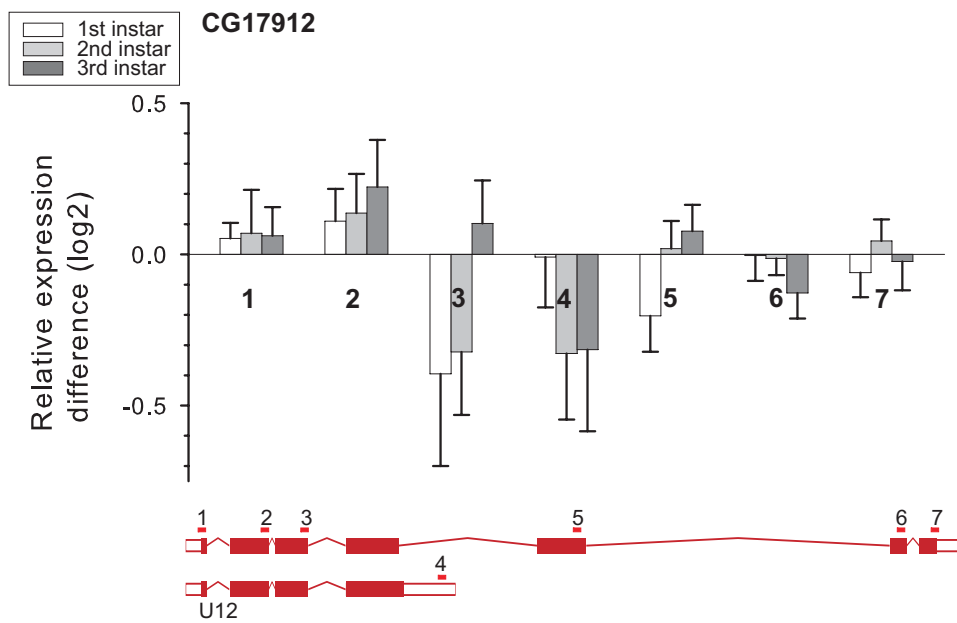

Supplement: Figure S1 — Expression changes in U12-type intron-containing genes. Plots depict probe-wise microarray results for upregulated (CG11984, CG34392, CG7736 and CG18177), downregulated (CG33108) and nonsignificant (CG7892, CG8408, CG6323, CG3294, CG15735, CG16941, CG17228, CG11839 and CG17912) U12 intron genes. Similar plots for CG34449, CG15081 and CG11328 are shown in Fig. 4. CG15899 and CG4894 were not plotted due to a large number of exons. Error bars indicate standard deviation. Significant changes at p<0.01 are marked with ‘*’. Schematic drawings of the most common splicing isoforms that indicate the positions of individual probes (horizontal bars and probe numbers above the exons) and U12-type introns are shown below each bar chart. Filled rectangles indicate exons of the coding sequence and open rectangles UTR regions. Introns are indicated as lines; the dotted lines indicate large introns that have been truncated in the schematic picture. (0.25 MB PDF) [file pone.0013215.s001.pdf]
